# Implementation of a Follow-Up Program for Intensive Care Unit Survivors

**DOI:** 10.3390/ijerph181910122

**Published:** 2021-09-26

**Authors:** Marta Villa, Silvia Villa, Simona Vimercati, Mara Andreossi, Fabrizia Mauri, Daniela Ferlicca, Roberto Rona, Giuseppe Foti, Alberto Lucchini

**Affiliations:** General Intensive Care Unit, Emergency Department—ASST Monza—San Gerardo Hospital, University of Milano-Bicocca, Via Pergolesi 33, 20900 Monza, Italy; marta.villa418@gmail.com (M.V.); silviavilla77@gmail.com (S.V.); simona.vimercati@asst-monza.it (S.V.); marazica@libero.it (M.A.); fabriziamauri@yahoo.it (F.M.); daniferlicca@gmail.com (D.F.); roberto.rona@libero.it (R.R.); g.foti@asst-monza.it (G.F.)

**Keywords:** 6-min walking test, follow-up, health related quality of life, ICU survivors, post-traumatic stress disorder

## Abstract

In Intensive Care Unit (ICU) survivors, critical illness has an impact on an individual’s long-term health status and quality of life. Adults who have recovered from intensive care management could develop muscle weakness, neurocognitive impairment, difficulties in managing activities of daily living and to returning to work, and psychological problems such as depression and anxiety. A prospective, observational study was designed. Data were collected from January to December 2018 from a structured follow-up program, at 3 time points after ICU discharge: at seven days, a visit in the general ward, a phone interview at three months and an ambulatory visit at six months. A total of 95 patients were enrolled, 36% female, with a median age of 65 (55–73) years and a median ICU length of stay of 13 (8–20) days. At the seven days follow-up, patients who had a positive Hospital Anxiety and Depression Scale showed a significantly longer time of intubation (*p* = 0.048) and length of ICU stay (*p* = 0.023). At three months, we observed a significant relationship between a positive Hospital Anxiety and Depression Scale and a median value of EuroQol-5D (*p* = 0.048). At six months, we observed that patients who had a positive Post-Traumatic Symptom Scale were significantly younger than the other group. Findings from the present study suggest that a longer time of intubation and length of ICU stay are associated with a higher level of anxiety and depression immediately after ICU discharge. Follow-up programs are recommended to assess and rehabilitate cognitive function at ICU discharge.

## 1. Introduction

The Intensive Care Unit (ICU) is a medical facility equipped with advanced technologies and personnel trained to provide intensive, advanced life-supportive care to critically ill patients. Although ICU mortality rates depend on severity of illness, co-morbidities, and diagnosis, among other factors, patients admitted to ICU have a high risk of mortality after hospital discharge [1,2]. For many years, the primary outcome of ICU studies was the survival or mortality rate, indicators easy to measure. Advances in ICU treatments have increased the number of survivors who require specialized care for ICU-related sequelae [3,4]. The impact of critical illness upon physical and mental health post-ICU has been well investigated. The risk of developing psychological disability after discharge from ICU ranges from one to sixty-two percent in the form of depression, anxiety, and post-traumatic stress disorder (PTSD) [4,5,6]. The risk factors are same as for cognitive impairment and include the female gender, lower education level, preexisting disability, and the use of sedation and analgesia in ICU [7,8]. PTSD is a reactive disturbance of the psychological sphere, frequently reported as a response to an heavy physical and/or mental stress. However best practices for specialized clinic settings, modes of treatment, and efficacy of follow-up care for prevalent ICU sequelae remain under-explored and under-reported [8].

Problems known as post intensive care syndrome (PICS), that are experienced by ICU survivors, appear to persist over time with a possible negative impact on health-related quality of life (HRQoL) [9]. The common symptoms include anxious or depressed mood, generalized weakness, decreased mobility, fatigue, sleep disturbances, sexual dysfunction, and cognitive issues (memory disturbance/loss, slow mental processing, poor concentration, and so on) [10]. Vulnerability and individual adapting capacity (resilience) play a fundamental role in the development and severity of acute stress reactions. Delirium with associated frightening psychotic experiences and post-ICU memories of in-ICU experiences are strong predictors of PTSD [11]. Delirium is difficultly recognized by clinicians and, when it is noted, it is often considered an “expected” occurrence attributed to ICU psychosis. Ely et al. described delirium in mechanically ventilated adult patients, alert or easily aroused, who are assumed to be cognitively intact by ICU personnel [12]. The most common type of delirium, hypoactive or quiet delirium may be associated with a worse prognosis than hyperactive or agitate delirium [10,11,12].

Focus on HRQoL after ICU recovery is another important outcome to plan and provide adequate answers to patients’ real needs after critical care and hospital discharge.

The fusion of physical, cognitive, and psychological symptoms in PICS can profoundly limit the social integration of ICU survivors. At one year, approximately one-third never return to paid employment, and those who do frequently report a reduction in income [13] More than 30% of ICU survivors require daily caregiver assistance, and many are unable to drive, limiting community engagement [14]. Quality of life is diminished following critical illness, highlighting the importance of optimal rehabilitation [15,16]. Knowing the consequences of an ICU stay could also help clinicians, nurses and all hospital staff to apply integrated preventive measures to improve outcomes in terms of quality of care.

Traumatic memories, PTSD diagnosis and evaluation of HRQoL should be, therefore, included as significant end points in studies evaluating the outcome of intensive care [8,9,10]. A healthcare model could provide the necessary services specific to ICU survivors’ healthcare needs. Specific ICU follow-up services are relatively recent developments in health systems, and may have the potential to address PICS through targeting unmet health needs arising from the experience of an ICU stay [17,18,19].

## 2. Aims of the Study

The first aim of this study is to evaluate the results, one year after the implementation of a structured follow-up program for ICU discharged patients in terms of incidence of PTSD, anxiety and depression. Secondary objectives are to measure the relationship among Post-Traumatic Stress Disorder anxiety, depression symptoms and the following items: age, length of ICU stay, days of mechanical ventilation, presence of delirium, pharmacological treatments and sedatives drugs used during ICU stay.

## 3. Materials and Methods

### 3.1. Study Design and Observed Variables

A prospective, observational cohort study was implemented. At San Gerardo University hospital, since January 2018, patients were invited to attend a follow-up program, after ICU discharge. Data were collected for 1 year of time (patients admitted from January to December 2018), enrolling all adult patients (age > 18 years) with ICU length of stay ≥96 h, mechanical ventilation and/or infusion of vasoactive drugs. The study was performed in a general ICU and ECMO centre of an Italian University Hospital. The ICU is an eight-bed unit (4 rooms with two beds), operating 24 h a day -/7 days a week plus two beds dedicated for post-surgical patients, available from Monday morning to Saturday afternoon. These two beds, dedicated to patients undergoing elective surgery with a planned overnight stay in ICU, were not included in the project.

The follow-up team consisted of three ICU nurses and two ICU physicians, who underwent a dedicated training course. 

### 3.2. Follow-Up Program

The follow-up program was structured in 3 time points for every patient enrolled: 

Ward visit one week after ICU discharge: An interview to evaluate the presence of delirium using Confusion Assessment Method for Intensive Care Unit (CAM-ICU) scoring was performed in the hospital ward as a first step, by ICU nurses [20,21]. Second, if CAM-ICU results were negative, the Post-Traumatic Symptoms Scale-10 (PTSS-10 scale) [22,23] and the Hospital Anxiety and Depression Scale (HADS) [24,25,26,27] scale were administered to identify PTSD, anxiety and/or depression symptoms. Activities of daily living performance were assessed using the Barthel Index [28,29,30]. If patients had already been discharged from the ward, these scales were tested by phone call. Visit and phone call have been conducted by trained ICU nurse.

Three months after ICU discharge: Multidimensional outcomes were evaluated through the phone administration of various tests, performed by a trained ICU nurse, to assess health status: PTSS-10 scale, HADS) scale and Barthel Index, and EuroQol-5D scale (EQ-5D) to assess HRQoL [31,32,33].

FU six months after ICU discharge: An ambulatorial visit was performed by an anesthetist and trained ICU nurse to assess the health status, Post Traumatic Stress Disorder, anxiety and/or depression symptoms, activities of daily living performance. In addition to the test performed at three months FU phone interview, patients were asked to perform a “six minutes walking test” (6MWT) [34] and an ambulatory spirometry.

### 3.3. Instruments

The selection of tools was based on the recommendations from the Improve Long Term Outcome Research after Acute Respiratory Failure work [35]. 

The CAM-ICU [22,23] uses objective assessments with prespecified cut-offs to determine the presence of inattention and disorganized thinking. To test for inattention, the CAMICU uses the Attention Screening Examination, which has auditory and visual components. The auditory component uses the Vigilance A letter test, which asks the patient to squeeze every time he or she hears the letter “A” 33; a series of 10 letters (“SAVEAHAART”) is given every 3 s. The visual component uses a picture recognition test; the patient is initially shown five simple pictures at 3-s intervals. Then the patient is shown 10 pictures and must identify which pictures were seen previously. If a patient makes three or more errors on the ASE letter or picture component, then the patient is considered to be inattentive. If the patient is unable or refuses to perform either ASE component, then the patient is also considered to be positive for inattention. To assess for altered level of consciousness, the CAM-ICU uses the Richmond Agitation Sedation Scale (RASS) to quantify altered level of consciousness. This scales ranges from –5 (comatose) to +4 (combative). A patient with a RASS other than 0 (alert, normal level of consciousness) is considered to be positive. To test for disorganized thinking, the rater asks four yes or no questions and asks the patient to perform a simple command. A patient who makes two or more errors is considered to have disorganized thinking. The CAM-ICU is positive if a patient has both altered mental status or fluctuating course and inattention and either altered level of consciousness or disorganized thinking.

The PTSS-10 is a validated and reliable screening tool to quantify traumatic memories and to measure and detect PTSD-related symptoms for the diagnosis of PTSD among ICU survivors. It consists of 10 questions concerning ongoing stress symptoms. Each item is scored from 1 (never) to 7 (always) with a total score range from 10 to 70 points. A score above 34 indicates clinically significant post-traumatic stress symptoms and is associated with a diagnosis of PTSD [22] We used the Italian version [23].

The HADS is a scale to detect states of anxiety and depression. The HADS is a questionnaire consisting of two subscales measuring patients’ symptoms of anxiety and depression. Each subscale consists of seven items scored from 0 to 3, resulting in a subscale score range from 0 to 21. A subscale score above 7 suggests clinically significant problems. The questionnaire has been validated among general medical patients as well as critically ill patients [24,25]. The HADS has good internal consistency and a 2-factor structure (anxiety and depression) in ICU survivors [26]. It is a reliable and valid instrument for assessing anxiety and depression in medical patients. Its construction facilitates its use with these patients. We used the Italian version of HADS [27]. The two 7-item scales were: one for anxiety and one for depression. We used the total scale to identify patients with emotional disorders who could benefit from a more specific psychiatric diagnosis and an adequate intervention [24,25,27].

The Barthel Index (BI) is a tool to measure functional impairment. The 10-item form consists of 10 activities of daily living, including feeding, bathing, grooming, dressing, bowel and bladder control, toilet use, transfers (to chair and back), mobility, and stairs-climbing. Items are rated in terms of whether patients can perform the task independently, with assistance or are totally dependent (scored as 0, 5 or 10; 15 points per item for transfers and mobility). The total score is calculated by adding up the individual scores, and ranges from 0 (total dependence) to 100 (total independence) [28,29]. We used the Italian culturally adapted tool that as a whole has demonstrated to be valid, reliable, acceptable, easy to understand and rapidly administrable [30].

The EuroQol-5D scale (EQ-5D) self-report questionnaire (commonly known as EQ-5D) is an internationally developed health-related quality of life measure. It consists of the EQ5D descriptive system and a visual analogue scale (EQ-VAS). The descriptive system comprises five three-level items, representing various aspects of health: mobility, self-care, usual activities, pain/discomfort and anxiety/depression (mood). Each dimension has three levels: no problems (score 1), some problems (score 2), extreme problems (score 3). These scores result in a health profile. The EQ-VAS records the respondent’s self-rated health status on a vertical, 20 cm visual analogue scale where the endpoints are labelled ‘best imaginable health state’ (score 100) and ‘worst imaginable health state’ (score 0). This information can be used as a quantitative measure of health outcome as judged by the individual respondents [24,25]. We used the validated Italian version [33].

The six minutes walking test (6MWT) is a practical, simple test that measures the maximal distance that a patient can walk at his or her own pace in 6 min. This self-paced test is performed in an indoor corridor. The walking course should be 30 m long [33]. The 6MWT plays a key role in evaluating functional exercise capacity, assessing prognosis and evaluating response to treatment across a wide range of respiratory diseases [34]. During this test, we used a continuous oximetry connected with a tablet with Blue Night ^®^ program. 

### 3.4. Ethical Issue

Our institutional Ethics Committee approved this study (approval number: 138, 6 February 2020—Follow-up project). Written informed consent was obtained from all patients before ICU discharge. 

### 3.5. Statistical Analysis

We calculated and reported median, and quartile, and we adopted the Mann–Whitney test for their comparison. We expressed variables without normal distribution as median and interquartile ranges, and we compared them using the Mann–Whitney U-test. We constructed a frequency table, and we used the chi-square test or Fisher’s exact test for comparisons of proportions. We considered a two-tailed *p* < 0.05 as statistically significant. We used the SPSS software v. 25 for the statistical analysis.

## 4. Results

**Figure 1:**
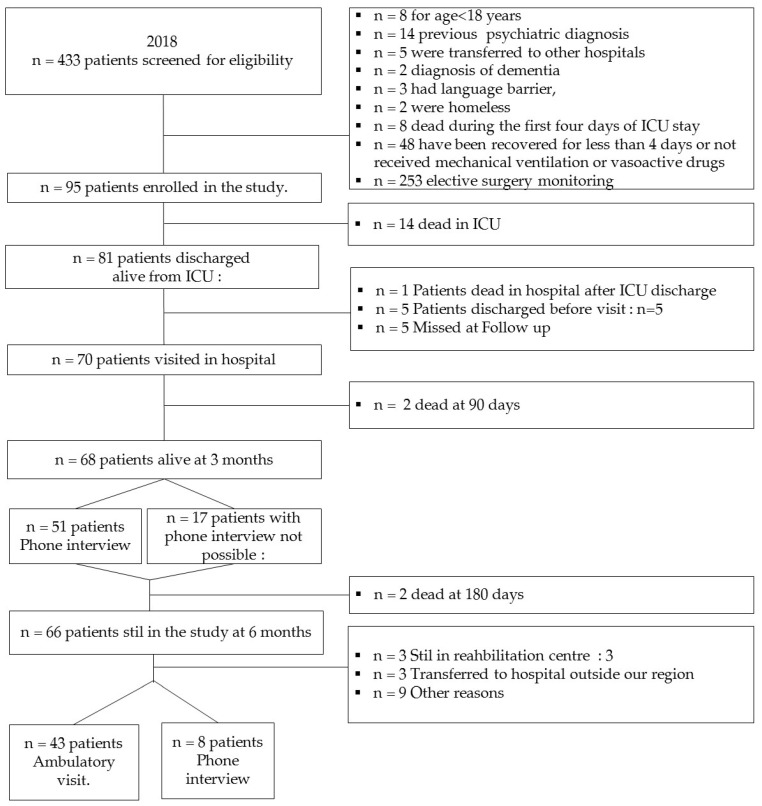
Patients’ enrolment following hospital discharge.

From 1 January to 31 December 2018, 433 patients were admitted to ICU, of which 95 (22%) matched the inclusion criteria for the FU program (Figure 1). The remaining 338 patients were excluded for the following reasons: *n* = 8 age < 18 years, *n* = 14 psychiatric previous diagnosis, *n* = 5 transferred to other hospitals, *n* = 2 diagnosis of dementia, *n* = 3 language barrier, *n* = 2 homeless, *n* = 8 died during the first four days of ICU stay, *n* = 48 less than 4 days of mechanical ventilation or vasoactive drugs an *n* = 253 elective post-surgery monitoring. 

Respiratory failure (*n* = 49–52.1%) was the most frequent diagnosis for ICU admission (*n* = 63–69%). As shown in Table 1, enrolled patients had a median age of 65 (IQR: 55–73) years, BMI of 27 (24–31) and Frailty Score of 2 (2–3) at ICU admission. Length of ICU stay (LOS) had a median value of 13 (8–20) days, intubation of 11 days (6–18) and sedation drug administration of 8 (4–15) days. A total of 42 (44%) patients were managed by neuro-muscular blocking agents (NMBA) for a median of 3 (1–7) days. In 15 patients (23%), prone positions were implemented, and 23 (16%) patients were submitted to Veno-Venous Extracorporeal Membrane Oxygenation (ECMO), for a median of 25 (14–27) days. During the ICU care, 15 patients of our sample died (16%). At seven days after discharge, 70 (86%) of survivors have been visited in general wards.

Delirium was detected in 7 patients. In the remaining patients, 63 patients tested negative for Delirium presence, median PTSS-10 was 10 (4–23) and median HADS was equal to 13 (9–18). At three months after ICU discharge, 68/70 (97%) patients were alive. Phone interviews were possible in 51 patients. Lower level depression and anxiety were identified, compared to the FU seven-days-visit, by PTSS-10 [median: 5 (1–11) vs. 10 (4–23, = 0.0023)] and HADS scale [median: 13 (9–18) vs. 4 (2–13), *p* = 0.04]. EuroQol-5D scale median value were equal to 75 (55–87). At six months 66 (95%) patients were still in the FU program. An FU visit by an anesthesiologist and ICU nurse was performed in 43 patients, and 8 patients were interviewed by phone because they were unable to come to hospital, due to distance or a clinical condition. Patients were seen in our outpatient clinic for a FU examination about six months after hospitalization. At sixth months after ICU discharge, patients have reached a good level of activities of daily living, assessed by Barthel Index, with a median score equal to 100 (80–100) and a good psychological status, with a median PTSS-10 equal to 7 (0–19), median HADS scale was 6 (3–14) and median EuroQol-5D scale was 80 (65–83).

A total of 28/43 (65%) patients performed the 6 Minute Walking Test (8 patients were unable to take the test and 5 patients performed the test during other medical check-up). The median distance walked in 6 min was 377 (277–413) meters, that is 72% (61–86) of the predicted value. During the test, none of the patients presented an arterial oxygen saturation <90%. Table 2 summarizes the data relating to the comparison between the three periods. At the 6-month follow-up 26 (60%) patients still had long-term side-effects related to ICU stay. The most relevant compromised domains of HRQoL were pain in two patients (8%), and depression/anxiety in three (12%), insomnia in seven (27%). Dysphagia was reported by 3 patients (12%). Medical device-related scarring was observed in 12 (28%) cases (VV-ECMO cannulas entry site, or tracheostomy). A total of 4 (9%) patients reported mild paraesthesia/limited mobility in the upper and lower limbs (2 patients in the left upper limb, and 4 patients in the lower limbs). The sample has been stratified for positive/negative results of tests (PTSS-10 ≥ 35, HADS ≥ 8) at three FU time points, analyzing the relation among days of sedation—days of intubation—LOS and age and EuroQol-5D scale. At the seven days follow-up, patients with a positive HADS showed a significantly longer time of intubation [median 6 (4–9) vs. 11 (6–19), *p* = 0.048] and LOS [median 8 (6–14) vs. 13 (8–27), *p* = 0.023]. At three and six months FU, these relations have not been maintained. At three months, we observed a significant relationship between positive HADS and median value of EuroQol-5D [median 85 (80–90) vs. 50 (50–70, *p* = 0.048]. At six months we observed that patients with positive PTSS-10 were significantly younger than the other group [median age: 60 years (53–67) vs. 44 (39–57), *p* = 0.030]. This small group of patients reached just sufficient level of HRQoL described by EuroQol-5D with a median value of 50 (50–100). Type of sedation, dose of sedatives and NMBA infusion was related to positive/negative results of tests (PTSS-10, HADS) at three FU time points, showing no significative differences.

## 5. Discussion

Post-Traumatic Stress Disorder as a result of an ICU stay had a median prevalence of 19% in ICU survivors [9,10]. ICU survivors enrolled in this study, mainly affected by respiratory failure, suggested that patients with longer LOS and intubation days developed positive HADS and PTSS-10 at FU seven days after ICU discharge. It is interesting to observe that anxiety and depression symptoms improved during the following months, as reported in previous studies [7,10]. At three and six months, most patients showed a reduction of this symptoms, a good level of HRQoL, expressed by the EuroQol-5D scale, with a nearly complete recovery of activity of daily living as shown by Barthel Index. Enrolled patients who had a positive PTSS-10 at six months were significantly younger than the other group. Furthermore, this small group of patients reached just the sufficient level of HRQoL described by EuroQol-5D. 

This could be explained by the different feeling about the quality of life in young people compared to older ones. It is likely that the ICU environment, the invasiveness of treatment and the isolation might have played a role in this result [1,7,9]. Although the Barthel Index suggested an almost complete functional recover after six months, younger people of working age referred to an inability to restart work, weakness, difficulty to focus on work activity, inability to concentrate and loss of employment [9,13]. These aspects could obviously influence the HRQoL. An additional sub-analysis was undertaken to investigate the relationship among type of sedative, use of neuromuscular blocking agents and incidence of PTSD and anxiety symptoms. No significant results were found due to the small sample size. 

At 6 months, most patients reported no problems in the mobility domain, likely a result of long-term rehabilitation and reintroduction to family life; no one reported issues with the ability to take care for themselves. In patients who underwent 6MWT, the average distance walked in 6 min was 377 (277–413) meters equal to 72% (61–86) of its predicted value and exertional desaturation or dyspnea were very uncommon. Patients who have recovered from critical illness frequently perform much worse, with a mean distance walked in 6 min of 361 (95%-confidence intervals 321–401) or 50–70% of the predicted value, 3 months after hospital discharge [4,33,34]. Other long-term complications due to ICU recovery, detected at the 6 months visit were as follows: Dysphagia reported by 3 patients (7%), medical device-related scarring by 12 (28%), paresthesia/limited mobility in the upper and lower limbs by 4 (10%). The incidence of dysphagia was similar to that reported by other studies [36,37,38], instead upper-limb nerve injuries in ICU survivors are very rare. In our sample, two patients reported neuropathic pain motor weakness on the left arm. Both patients were in ECMO and intermittently proned. Upper-limb nerve injuries related to brachial plexus neuropathy are also associated with the prone position [39,40,41,42]. 

Despite prone positioning has been shown to reduce mortality in ARDS patients [43,44], we must keep in mind that can also be associated with various complications, including pressure sores and accidental injuries [45,46,47,48]. During the study period, the prone position protocol in our unit involved the use of the “swimmer position”. In this position, our protocol required that only the left arm was raised [48]. For this reason, after an internal audit in which we discussed the follow-up results, we decided to avoid this position. Starting from January 2020, we don’t use the swimmer position during pronation. More investigations and retrospective studies are needed to define the exact prevalence of prone position complications.

Finally, a nurse-led follow-up after critical care was not a common activity in Italian intensive care units. Few ICU departments had a resource group organizing the follow-up activities. Internationally, the availability of ICU follow-up services is well documented, particularly in Northern Europe [49,50]. In our limited experience, implementing a nurse-led follow-up program requires a weekly commitment of approximately 8 h. 

## 6. Limitations and Future Lines of Research

Our study has some limitations which need to be addressed. 

The number of patients investigated was quite low. Due to the small number, it wasn’t possible to evaluate the effects of various therapies during the ICU stay. We could not compare our results with a patient baseline for pre-existing quality of life values as these were not available/not collected. (3) We performed only one 6MWT. Finally, a longer follow-up time is required to understand whether functional and psychological impairment is persistent or not.

The greatest advantage of the ICU follow-up program is its participatory development, which increases the likelihood of the intervention being effective. Though ICU follow-up clinics have existed for decades, few reports have been published of interventions and the effectiveness of interventions used in these clinics. Rigorous research is needed to establish standardized care for the growing population of ICU survivors.

## 7. Conclusions

In conclusion, our experience suggests that a longer intubation time and LOS are associated with higher level of anxiety and depression symptoms during the immediate discharging time. This trend improves in the following months with a reduction of these symptoms associated to a progressive recover of activity and daily living. In a small part of sample, including younger people, PTSD symptoms become consolidated at six months after ICU discharge, associated to worst level of HRQoL. We suggest implementing a follow-up program as an integral part of patient therapy during and after an ICU stay. This study shows how to organize a post-ICU nurse-led follow-up program, based on literature and expert opinion. Follow-up programs are recommended to assess and rehabilitate cognitive function at ICU discharge.

## Figures and Tables

**Table 1:** Baseline characteristics.

Study Population *n* = 95		Median (IQR)
Age (years)		65 (55–73)
Gender, Female *n* = (%)		34 (36%)
Weight Kg (at ICU admission)		75 (70–84)
Height (cm)		170 (164–175)
Body Mass Index		27 (24–31)
Frailty score		2 (2–3)
SOFA		8 (6–10)
SAPS 2		46 (37–54)
Type of patients	Medical	63 (69.2%)
	Surgical	18 (19.8%)
	Trauma	10 (11.0%)
Admitted to ICU from	Ward	16 (17.6%)
	Other ICU	32 (35.2%)
	Emergency room	43 (47.3%)
	Respiratory failure	49 (52.1%)
Major diagnosis for ICU admission	Sepsis	16 (17%)
	Cardiac arrest	5 (5.3%)
	Surgery	9 (9.6%)
	Non-septic shock	5 (5.3%)
	Trauma	7 (7.4%)
	Stroke	1 (1.1%)
Comorbidities	Chronic obstructive pulmonary disease	14 (15%)
	Heart failure	10 (11%)
	Renal failure	8 (8%)
	Hepatic failure	1 (1%)
	Cancer	11 (12%)
	Hematologic disease	8 (8%)
	Diabetes	14 (15%)
	Vasculopathy	8 (9%)
	Immunosuppression	10 (11%)
Length of ICU stay		13 (8–20)
Length of Hospital stay		30 (18–47)
Alive at ICU discharge		81 (85%)
Mechanical ventilation days		11 (6–18)
Patients with tracheostomy performed during ICU stay		21 (22%)
Veno-Venous Extracorporeal Membrane Oxygenation (yes)		16 (23%)
Continuous renal replacement therapy	(yes)	24 (25%)
Prone position (yes)		15 (16%)
Number of Prone Position cycles		3.5 (1.75–4)
Days of sedation drugs administration		8 (4–14)
	Propofol (intravenous)	51 (100%)
	Dexmetomidine (intravenous)	18 (19%)
	Isoflurane (volatile anaesthetic gas)	10 (11%)
Patients with oral sedation		54 (57%)
	Benzodiazepines	40 (74%)
	Quetiapine	9 (16%)
	Antidepressants	5 (10%)
NMBA infusion (yes)		42 (44%)
NMBA infusion days		5.5 (2–9)
Vasoactive drugs infusion		83 (87%)
Steroid administration		30 (32%)
Physiotherapist available during ICU stay		37 (39%)
CAM-ICU (positive at ICU discharge)		0 (0%)

Legend: SOFA: Sequential Organ Failure Assessment, SAPS 2: Simplified Acute Physiology Score, NMBA: neuro muscular blocking agents, CAM-ICU: Confusion Assessment Method-Intensive Care Unit.

**Table 2:** PTSS-10, HADS and EQ-VAS modifications at 7 days, 3 and six months after ICU discharge.

	7 Days Hospital Visit	3 Months Phone Interview	6 Months Hospital Visit or Phone Interview	*p*. Value
Alive	*n* = 70	*n* = 68	*n* = 51	
CAM-ICU (positive)	6 (9%)	---	---	
PTSS-10—median (IQR)	10 (4–23)	5 (1–11)	7 (0–19)	0.004
Pts with PTSS-10 ≥ 35	5 (7%)	2 (4%)	4 (9%)	0.006
HADS—median (IQR)	13 (9–18)	4 (2–13)	6 (3–14)	0.0001
Pts with HADS ≥ 8	47 (70%)	20 (40%)	20 (46%)	0.05
EuroQol-5D-VAS median (IQR)	---	75 (55–87)	80 (65–83)	0.441

Legend: CAM-ICU: Confusion Assessment Method-Intensive Care Unit, PTSS-10: Post-Traumatic Symptoms Scale-10 (cut-off ≥ 35), HADS: Hospital Anxiety and Depression Scale (cut-off ≥ 8), EuroQol-5D-VAS: respondent’s self-rated health status on a vertical 20 cm visual analogue scale.

## Data Availability

Data are available on request due to ethical restrictions.
